# Treatment of Bartholin gland cyst with CO_2_ laser

**DOI:** 10.1590/S1679-45082016AO3568

**Published:** 2016

**Authors:** Neila Maria de Góis Speck, Karol Pereira Ruela Boechat, Georgia Mouzinho Lima dos Santos, Julisa Chamorro Lascasas Ribalta

**Affiliations:** 1 Escola Paulista de Medicina, Universidade Federal de São Paulo, São Paulo, SP, Brazil.

**Keywords:** Bartholin’s glands/pathology, Vulvar vestibulitis/therapy, Lasers, gas/therapeutic use

## Abstract

**Objective:**

To describe the results of treatment with CO_2_ laser for Bartholin gland cysts.

**Methods:**

Thirty-one women with Bartholin gland cysts were treated with CO_2_ laser at an outpatient´s setting. Skin incision was performed with focused laser beam, the capsule was opened to drain mucoid content, followed by internal vaporization of impaired capsule.

**Results:**

There were no complications. Five patients had recurrence of the cyst and were submitted to a second and successful session.

**Conclusion:**

CO_2_ laser surgery was effective to treat Bartholin gland cysts with minimal or no complications, and can be performed at an outpatient´s setting.

## INTRODUCTION

Bartholin glands, or greater vestibular glands, are located one on each side of the labia minor, externally to the vaginal opening. During sexual stimulation, these glands release lubricant fluid.^1^

The first person to describe this gland was Kaspar Bartholin, in 1977. This Dane became known for describing it along with the accessory ducts of the salivary and sublingual glands.^1-3^

The Bartholin gland cyst consists of a tumor-like lesion of the vulva, which develops along the trajectory of the gland duct; despite its obstruction, the gland continues to secrete mucus. This situation accounts for 2% of gynecological appointments per year of women at child-bearing age.^2^

A study performed in Korea in 2009 reported that the incidence of cysts and abscesses of Bartholin gland occurred in women aged 15-50 years, with a greater incidence up to 40 years, decreasing from then on.^4^

In Brazil, in an article published in 2012, the mean age of 127 women with Bartholin gland cysts was 37.3 years, ranging from 18 to 61 years. Approximately 70% were multiparous, and the most frequent complaint was pain.^5^

Although benign, the lesion is associated with significant discomfort for patients. The most commonly described symptom is local pain, which may worsen when walking or sitting. If the cyst evolves to an abscess, the symptoms may be more severe and incapacitating, and even be accompanied by nausea, vomiting, and fever, similar to an infectious condition.^6^

The obstruction of the Bartholin gland originates retention of secretions with duct dilation and cyst formation. Oftentimes, women remain asymptomatic. However, if the cyst is infected and an abscess develops, the clinical picture becomes symptomatic. Diagnosis is made by means of the physical examination, observing a fluctuating mass in one of the small labia.^3^

Bartholin gland abscesses are common in single women and those of lower social and economic conditions.^1^ Some studies showed that the secretion originated in these abscesses presented with agents such as *Neisseria gonorrhoeae* and *Chlamydia trachomatis*, but in most cases, the causative organisms are from the mixed vaginal flora, bacteroides*, Escherichia coli,* and *Staphylococcus aureus*.^1,3^

The differential diagnosis of the Bartholin gland cysts and abscesses is made with other vulvar masses, such as epidermal inclusion cysts, and cysts of Nuck and Skene duct. Carcinoma is rare. A study performed in Warsaw, Poland, from 1980 to 2009, recorded 1,296 patients with vulvar carcinoma treated at an oncology center. Nine of them had carcinoma of the Bartholin gland, including three patients with squamous cell carcinoma, three with cystic adenoid carcinoma, and three with sarcoma.^7^

Treatment used for cysts and abscesses of the Bartholin gland is still very controversial. There are many options, including antibiotics, drainage, marsupialization, gland excision, and destruction or cauterization of the cyst with silver nitrate and CO_2_ laser.^6^

### Therapeutic options

The treatment of Bartholin cyst may be conservative or surgical, depending on the patient’s symptoms, size of the cyst, and whether it is infected or not. Asymptomatic patients require no treatment. If the gland becomes infected, treatment with broad-spectrum antibiotics and analgesics is necessary.^3,8^

The gold standard treatment is surgical removal of the entire cyst. However, this treatment is not performed very often since it affects the physiological vaginal lubrication and is associated with other complications. Less invasive therapeutic strategies have been proposed.^6,8^

### Marsupialization

This was initially described by Jacobson, in 1950. It is performed with a small 1.5-to-3cm incision over the abscess to minimize scars and allow drainage of the gland’s secretion. After drainage, the capsule of the cyst is sutured with the borders fixed to the exterior, in order to avoid closing and formation a new cyst; over time, the process undergoes reepithelization. The cavity should be irrigated with saline solution and an antibiotic. This procedure is normally done under anesthesia - either general, local, or pudendal nerve block.^1,3^

In a randomized prospective study, 83 women submitted to marsupialization were followed up, 24.1% presented with recurrence, and the most frequent postoperative symptoms were discharge at the surgical site and labial edema.^9^Studies on marsupialization reported a global recurrence rate varying from 2 to 25%.^10^

It is important to point out that if the cyst develops in a postmenopausal woman, a biopsy should be done to evaluate possible malignity.^11^

### Fistulization – Word catheter and Jacobi ring

Placement of the Word catheter is a technique first described by Buford Word, in 1964, and has the advantages of being a simple method, it is possible to perform the procedure at an outpatient setting, and patients recover promptly. The disadvantage is no availability of this catheter at hospitals. The success of this method is based on the ancient principle that a foreign body in a wound hinders its natural closure, resulting in the formation of a fistula with wound reepithelization.^3,12^

Word catheters are placed by means of a 5mm incision inside the small labia, in the region of the Bartholin gland. At the extremity of the catheter, there is a sac that is inflated with up to 3mL of sterile saline solution, and the catheter is left in place for 4 to 6 weeks.^6^

A study published in 2008 developed a technique similar to that of the Word catheter. It proposed the placement of a device similar to this catheter that allowed drainage and reepithelization, and was removed after three weeks with total recovery of the patient.^12^

The Jacobi ring is a rubber catheter that can be inserted through the cyst or abscess and secured by suture. This device also allows drainage and reepithelization.^13^

In a randomized study comparing the Word catheter and the Jacobi ring, there were no differences in recurrence rates in both techniques, but the authors showed greater patient satisfaction with the use of the ring.^14^

Recurrence of Bartholin cyst was observed in 4 to 17% of 111 women after use of the catheter during a six-month follow-up. Premature loss of the catheter was the most common adverse event.^14^

### Ablation with silver nitrate

Application of silver nitrate to treat Bartholin gland cyst or abscess is considered a simple and effective technique, which can be performed in an outpatient setting. After local cleaning and anesthetic infiltration, a 0.5-to-1.0cm long incision is made on the mucosa over the cyst, the content is drained and silver nitrate is placed on the cavity, with no sutures. The solution is removed 48 hours later.^3^

In a study done with a group of 76 patients submitted to this treatment, the recurrence rate was 26.3%. The most frequent postoperative complaints were a burning sensation at the surgical site, hematoma, and dyspareunia.^9^

### Sclerotherapy with alcohol

This is a procedure carried out in a short amount of time and with a rapid rate of cure. After incision of the skin and drainage of the cyst emptying the cavity, the site is irrigated with 70% alcohol for approximately 5 minutes. Recurrence was observed in 8 to 10% of cases, and the most common postoperative sign was transient hyperemia.^15^

### Bartholinectomy

Exeresis of Bartholin gland is a surgical treatment that requires greater operative time. It is an elective procedure, performed in the absence of infection, and it is normally used after failure of other techniques.^1,6^

### CO2 laser

CO_2_ laser can be use both to vaporize and to remove the Bartholin gland. This surgical procedure is simple and quick, but expensive. It may be performed at an outpatient setting, with minimal discomfort for the patient in the intra- and postoperative periods.^8^

A study reported a series of 19 patients with Bartholin cysts treated with the minimally invasive approach of CO_2_ laser, and demonstrated the surgical procedure as being extremely simple, and performed in a very short time, on average, seven minutes. Patients’ satisfaction was high in the short- and long-run.^16^

The objective of the study done by Panici et al. was to describe the conservative surgical technique with CO_2_ laser, evaluate its viability, complication rate, and the results obtained. It demonstrated that laser treatment can be safely used at the outpatient setting, patients complained very little of intraoperative pain, and there are high rates of cure in the long-term.^16^

The analysis of 200 patients with Bartholin cysts submitted to CO_2_ laser treatment showed a mean age of 32 years, with one delivery, and 87% of patients received some antibiotic therapy. As to the follow-up data after the procedure, the rate of cure with a single laser application was 95.7%, and repeating laser application was effective in cases of recurrence during follow-up.^17^

The study published by Speck et al. evaluated the use of laser in 22 women. The patients were oriented to return two and four weeks after the procedure for follow-up. At the first visit, all presented with mucoid discharge, and complete recovery occurred within a period of 3 to 4 weeks. Only two patients presented with recurrences, requiring a new application.^18^

This form of treatment seems to be a good alternative, less invasive, quick, and safe for cases of Bartholin cysts. The recurrence rates, on average, were less than 10% and cure after a new laser procedure is complete.^16,18^

## OBJECTIVE

To describe the results of treatment with CO_2_ laser for Bartholin gland cyst.

## METHODS

During the period from January 2007 to December 2014, 31 patients diagnosed with Bartholin gland cysts were seen at the Unit for Nucleus for Prevention of Gynecological Disease - Department of Gynecology, *Escola Paulista de Medicina*. This study was approved under CAAE: 45073715.3.0000.5505. A retrospective evaluation was made of the medical records of 31 patients, and the data for description of the technique, along with the results, were grouped.

The mean age of the group was 34.9 years, ranging from 22 to 48 years.

Four patients had already been treated; three of them used antibiotic treatment and one of them underwent bartholinectomy.

### Surgical procedure

**Figure 1:**
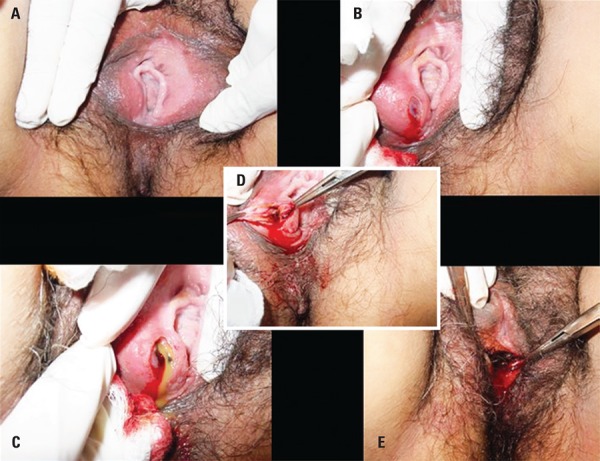
Procedure with CO2 laser in the treatment of a Bartholin cyst. (A) Exposure of the cyst to the right, with 2% xylocaine infiltration. (B) Incision with the focused laser beam over the cyst. (C) Opening of the capsule with complete drainage of the content. (D) Repair of the borders with exposure of the internal surface of the capsule. (E) Vaporization of the internal capsule with unfocussed laser beam

The procedure consisted of antisepsis with a solution of topical povidone-iodine and local anesthesia with 2% xylocaine with a vasoconstrictor. With the CO_2_ laser at the potency of 10 to 25W, used continuously coupled to the colposcope, a longitudinal incision was made with the laser beam using a focused beam to open the capsule of the cyst. The lateral borders of the incision were maintained under tension with grippers for exposure of the cavity, with subsequent drainage of the content and cleaning of the interior with sterile saline solution. Destruction of the capsule tissue was performed with vaporization, using the unfocused laser beam (Figure 1). Patients were instructed to do sitz-baths with a solution of povidone-iodine diluted in water, three times a day, and to abstain from sexual activity for 2 to 3 weeks. Antibiotics and analgesics were prescribed for patients presenting any sign of infection.

## RESULTS

The procedure was performed with local anesthesia at an outpatient clinic. All patients reported minimal discomfort, inherent to outpatient’s procedures, with pain with the anesthetic infiltration and sensation of local heat with the application of the laser beam.

The patients were evaluated 15 and 30 days after the laser treatment. In the first two weeks, there was continuous drainage of mucus, and at the end of the first month, there was complete healing of the surgical wound.

Five patients (16%) experienced Bartholin cysts recurrence until 6 months after the first laser session and were submitted to a second session. After the second phase of treatment, there were no recurrences.

In the 31 cases analyzed, after the healing period, there were no reports of pain at the incision site, and there were no scars and retractions.

## DISCUSSION

In Italy, this technique has been used by several authors, due to the ease of access to laser technology.^8,16,17^ In Brazil, we note very little experience of laser use for marsupialization of the Bartholin cyst due to cost of the equipment.^5^ Our organization, focused primarily on teaching in the medical field, is considered a reference in care of lower genital tract diseases. Experience with the said technique was due to the fact of having the laser equipment, and the great number of patients with the said disease. In a prior analysis of our cases, in 2007, we had already noted positive responses to this surgical method.^18^

The recurrence rate in our survey was similar to that of literature, according to the various techniques initially described. Treating the Bartholin cyst with CO_2_ laser proved effective for resolution of the disease and of post–treatment comorbidities, such as pain and scars in the affected area.^18^ It is a treatment that associates marsupialization with the destruction of the diseased capsule by means of the laser bean, providing good results, albeit no different from those that use only surgery or cauterization.^1,3,6,16^

The procedure was performed at an outpatient clinic, with a minimal amount of bleeding during the act, besides good and rapid healing. Surgical methods with a cold scalpel are not exempt from causing greater bleeding, and those destructive with cauterization by means of alcohol or silver nitrate can be more painful in the postoperative phase. These are factors that have influenced our preference for the technique presented.^1,3,16^

The CO_2_ laser proved to be an effective method with low morbidity. We noted, with this technique, that healing was rapid, with minimum fibrosis and very little pain, a complication seen with the conventional techniques described by other authors.^3,4,5^

These women are generally young, sexually active and we should be concerned with the frequent risk of sequelae, such as dyspareunia due to fibrosis of the gland, especially with the destructive cauterization approaches.

Additionally, this technique allows new sessions in case of a recurrence.

Since this is an outpatient method, the patient does not need to be away from her activities for very long, there is no need to be admitted to hospital, and there is a reduction in hospital costs. On the other hand, the equipment is very expensive and requires a qualified professional to handle it.

Since there is no hospitalization, the patient is discharged home after the procedure, and soon returns to her daily activities.

## CONCLUSION

The Bartholin cyst approach with CO_2_ laser, according to the technique described of opening of the cyst, drainage of the content, and vaporization of the capsule, can be used as an effective conservative treatment with low rates of recurrence, complications, and great patient satisfaction. It does not require hospital admission, which means saving money and time. The technique to be used depends on the skills of the physician and availability of equipment.
