# Supplementary material for: Treatment of Bartholin gland cyst with CO2 laser
Source: Einstein (Sao Paulo). 2016 Jan-Mar;14(1):25–9. doi: 10.1590/S1679-45082016AO3568 (PMC4872913; doi:10.1590/S1679-45082016AO3568)
Supplement: Translation

# Translation: Tratamento do cisto da glândula de Bartholin com *laser* de CO_2_

**Authors:** Neila Maria de Góis Speck, Karol Pereira Ruela Boechat, Georgia Mouzinho Lima dos Santos, Julisa Chamorro Lascasas Ribalta

**Affiliations:** 1 Escola Paulista de Medicina, Universidade Federal de São Paulo, São Paulo, SP, Brasil.

**Keywords:** Glândulas vestibulares maiores/patologia, Vestibulite vulvar/terapia, Lasers de gás/uso terapêutico

## Abstract

**Objetivo:**

Descrever os resultados do tratamento com *laser* de CO_2_ para o cisto da glândula de Bartholin.

**Métodos:**

Foram tratadas 31 mulheres com cisto da glândula de Bartholin em regime ambulatorial com o *laser* de CO_2_. Foram realizados: incisão da pele com feixe de *laser* focalizado, abertura da cápsula com drenagem do conteúdo mucoide e vaporização interna da cápsula doente.

**Resultados:**

Não ocorreram complicações. Cinco pacientes apresentaram recidiva do cisto, sendo submetidas à segunda sessão, com sucesso em todas.

**Conclusão:**

A cirurgia com *laser* de CO_2_ foi efetiva para o tratamento do cisto da glândula de Bartholin, com mínima ou nenhuma complicação, podendo ser realizada em ambiente ambulatorial.

## INTRODUÇÃO

As glândulas de Bartholin, ou glândulas vestibulares maiores, estão localizadas uma de cada lado interno dos lábios menores, externamente à abertura vaginal. Durante a estimulação sexual, essas glândulas liberam fluido lubrificante.^1^

O primeiro a descrever a glândula foi Kaspar Bartholin, em 1977. Esse dinamarquês ficou conhecido por descrevê-las, bem como os ductos acessórios das glândulas salivares e sublinguais.^1-3^

O cisto de glândula de Bartholin consiste em tumoração da vulva, que se desenvolve no trajeto do ducto glandular por sua obstrução, e a mesma continua a secretar muco. Essa situação é responsável por 2% das consultas ginecológicas ao ano em mulheres na idade reprodutiva.^2^

Estudo realizado na Coreia em 2009 relatou que a incidência de cistos e abcessos da glândula de Bartholin ocorria em mulheres entre 15 e 50 anos, com maior incidência até os 40 anos e decréscimo a partir dessa idade.^4^

No Brasil, em publicação de 2012, a média etária descrita das portadoras de cisto de Bartholin foi de 37,3 anos, variando de 18 a 61 anos, no total de 127 mulheres. Aproximadamente 70% eram multíparas, e a queixa mais frequente foi a dor.^5^

Embora benigna, a lesão está associada a desconforto significante das pacientes. O sintoma mais descrito é dor local, que pode piorar ao deambular ou sentar. Se o cisto evoluir para abcesso, os sintomas podem ser mais severos e incapacitantes, e estarem acompanhados de náuseas, vômitos e febre, semelhantemente a um quadro infeccioso.^6^

A obstrução da glândula de Bartholin dá origem à retenção de secreções com dilatação do ducto e formação do cisto. A mulher, por muitas vezes, pode ficar assintomática com sua presença. Porém, se o cisto for infectado e desenvolver abscesso, o quadro torna-se bastante sintomático. O diagnóstico é realizado por meio do exame físico, observando-se massa flutuante em um dos pequenos lábios.^3^

Abcessos da glândula de Bartholin são mais comuns em mulheres solteiras e de origem socioeconômica mais baixa.^1^ Alguns estudos mostraram que a secreção originada desses abscessos apresentava agentes como a *Neisseria gonorrhoeae* e *Chlamydia trachomatis*, porém, na maioria dos casos, os organismos causadores são da flora vaginal mista, bacteroides*, Escherichia coli e Staphylococcus aureus*.^1,3^

O diagnóstico diferencial dos cistos e abscessos da glândula de Bartholin é feito com outras massas vulvares, como cistos de inclusão epidérmica, cistos de canal de Nuck e Skene. O carcinoma é raro. Estudo realizado em Varsóvia, na Polónia, nos anos de 1980 a 2009, registrou 1.296 pacientes com carcinoma vulvar que foram tratados em centro de oncologia. Nove delas tiveram carcinoma da glândula de Bartholin, incluindo três pacientes com carcinoma espinocelular, três com carcinoma adenoide cístico e três com sarcoma.^7^

O tratamento usado para cistos e abscessos da glândula de Bartholin ainda é muito controverso. Há muitas opções para tratamento, incluindo antibióticos, drenagem, marsupialização, excisão da glândula, e destruição ou cauterização do cisto com nitrato de prata e *laser* de CO_2_.^6^

### Opções terapêuticas

O tratamento do cisto de Bartholin pode ser conservador ou cirúrgico, dependendo dos sintomas da paciente, do tamanho do cisto, e se está infectado ou não. Pacientes assintomáticas não necessitam de qualquer tratamento. Se a glândula torna-se infectada, o tratamento com antibióticos de amplo espectro e analgésicos faz-se necessário.^3,8^

O padrão-ouro de tratamento é a remoção cirúrgica de todo o cisto. No entanto, este tratamento muitas vezes não é realizado porque afeta a lubrificação vaginal fisiológica e está associado a outras complicações. Estratégias terapêuticas menos invasivas têm sido propostas.^6,8^

### Marsupialização

Foi inicialmente descrita por Jacobson, no ano de 1950. Realizada com uma pequena incisão de 1,5 a 3cm sobre o abscesso, para minimizar cicatrizes e permitir a drenagem de secreções da glândula. Após a drenagem, faz-se sutura da cápsula do cisto com as bordas fixadas em seu exterior, para evitar o fechamento e a formação de novo cisto; com o passar do tempo, o processo sofre reepitelização. A cavidade deve ser irrigada com solução salina e antibiótico. Esse procedimento normalmente é realizado sob anestesia, que pode ser geral, local ou com bloqueio do nervo pudendo.^1,3^

Em estudo prospectivo randomizado, foram acompanhadas 83 mulheres submetidas à marsupialização, 24,1% apresentaram recidiva, e os sintomas pós-operatórios mais frequentes foram saída de secreção no sítio cirúrgico e edema labial.^9^

Estudos sobre marsupialização relataram taxa de recidiva global variando de 2 a 25%.^10^

É importante salientar que, se o cisto se desenvolver em mulher na pós-menopausa, a biópsia deve ser realizada, para ser avaliada possível malignidade.^11^

### Fistulização - cateter de Word e anel de Jacobi

A colocação de cateter de Word é técnica descrita pela primeira vez em 1964 por Buford Word e tem como vantagens a simplicidade do método, a possibilidade de colocação em meio ambulatorial e a fácil recuperação da paciente. A desvantagem é a falta desse cateter nas instituições hospitalares. O sucesso deste dispositivo é baseado no princípio antigo de que corpo estranho em uma ferida impede seu fechamento natural, resultando na formação de fístula com reepitelização.^3,12^

Os cateteres de Word são colocados por meio de incisão de 5mm no interior dos pequenos lábios, na região da glândula de Bartholin. Na extremidade do cateter, há uma bolsa que é insuflada com até 3mL de solução salina estéril, e o cateter é deixado no lugar por 4 a 6 semanas.^6^

Estudo publicado em 2008 desenvolveu técnica semelhante ao cateter de Word. Nele, foi proposta a colocação de dispositivo semelhante ao cateter, que permitia a drenagem e a reepitelização, sendo retirado após 3 semanas com total recuperação da paciente.^12^

O anel de Jacobi é um cateter de borracha que pode ser inserido por meio do cisto ou abscesso e fixado por sutura. Esse dispositivo também permite drenagem e reepitelização.^13^

Em estudo randomizado comparando o cateter de Word e o anel de Jacobi, não houve diferença nas taxas de recidiva nas duas técnicas, mas os autores mostraram maior satisfação das pacientes com o uso do anel.^14^

A recidiva do cisto de Bartholin foi observada em estudo com 111 mulheres, em 4 a 17% das pacientes após o uso do cateter no acompanhamento de 6 meses. A perda prematura do cateter foi o evento adverso mais comum.^14^

### Ablação com nitrato de prata

A aplicação de nitrato de prata para tratamento de cisto ou abscesso da glândula de Bartholin é considerada uma técnica simples, eficaz e que pode ser realizada em ambiente ambulatorial. Após a limpeza local e a infiltração de anestésico, é realizada incisão na mucosa sobre o cisto de 0,5 a 1,0cm de comprimento, drenagem do conteúdo e colocação de nitrato de prata na cavidade sem realização de suturas. A remoção da solução ocorre após 48 horas.^3^

Em estudo com um grupo de 76 pacientes submetidas a este tratamento, a taxa de recidiva foi de 26,3%. As queixas mais frequentes no pós-operatório foram queimação no sítio cirúrgico, hematoma e dispaurenia.^9^

### Escleroterapia com álcool

Trata-se de procedimento realizado em pouco tempo e com cura rápida. Após incisão de pele e drenagem do cisto com esvaziamento da cavidade, o local é irrigado com álcool 70% por aproximadamente 5 minutos. A recidiva foi observada em 8 a 10%, e o sinal mais comum no pós-operatório foi hiperemia transitória.^15^

### Bartolinectomia

A exerése da glândula de Bartholin é um tratamento cirúrgico que requer maior tempo operatório. Consiste em procedimento eletivo, realizado na ausência de infeção, normalmente utilizado após falha das outras técnicas.^1,6^

### Laser de CO2

O *laser* de CO_2_ pode ser utilizado tanto para vaporizar como para extirpar o cisto da glândula de Bartholin. Esse procedimento cirúrgico é simples e rápido, porém dispendioso. Pode ser realizado em ambiente ambulatorial, com mínimo desconforto da paciente no intra e pós-operatório.^8^

Estudo que relatou série de 19 pacientes com cisto de Bartholin tratadas com abordagem minimamente invasiva de *laser* CO_2_ demonstrou o procedimento cirúrgico como extremamente simples, sendo realizado em curtíssimo período de tempo, com média de 7 minutos. A satisfação das pacientes foi alta, a curto e longo prazo.^16^

O objetivo do estudo de Panici et al. foi descrever técnica cirúrgica alternativa conservadora com *laser* de CO_2_, avaliar a viabilidade, a taxa de complicação e os resultados obtidos. Demonstraram que tratamento com *laser* pode ser realizado com segurança em ambiente ambulatorial, pouca dor intraoperatória e altas taxas de cura a longo prazo.^16^

A análise de 200 pacientes com cisto de Bartholin submetidas ao tratamento com *laser* de CO_2_ mostrou que o perfil delas era média de idade de 32 anos, com um parto e 87% das pacientes receberam algum tipo de tratamento com antibióticos. Quanto aos dados de seguimento após o procedimento, a cura com uma única aplicação de *laser* foi de 95,7% e a repetição do *laser* foi efetiva nas recidiva durante o seguimento.^17^

O estudo publicado por Speck et al. avaliou o emprego do *laser* em 22 mulheres. As pacientes foram orientadas a retornar na segunda e quarta semana após o procedimento para seguimento. No primeiro controle, todas apresentavam drenagem de secreção mucoide, e a recuperação total ocorreu no período de 3 a 4 semanas. Apenas duas pacientes apresentaram recidiva, necessitando de nova abordagem.^18^

Essa forma de tratamento parece boa alternativa, menos invasiva, rápida e segura para cisto da glândula de Bartholin. As taxas de recidiva em média, não ultrapassam 10% e a cura após novo procedimento de *laser* é total.^16,18^

## OBJETIVO

Descrever os resultados do tratamento com *laser* de CO_2_ para o cisto da glândula de Bartholin

## MÉTODOS

No período de janeiro de 2007 a dezembro de 2014 foram atendidas 31 pacientes com diagnóstico de cisto da glândula de Bartholin no Núcleo de Prevenção em Doenças Ginecológicas do Departamento de Ginecologia da Escola Paulista de Medicina. Este estudo foi aprovado sob número do CAAE: 45073715.3.0000.5505. Foi realizada avaliação retrospectiva dos prontuários das 31 pacientes, os dados para descrição da técnica, com os seus resultados, foram reunidos.

A média etária do grupo foi de 34,9 anos, variando de 22 a 48 anos.

Quatro pacientes já tinham sido tratadas, sendo que três utilizaram antibioticoterapia e uma, bartolinectomia.

### Procedimento cirúrgico

O procedimento consistiu na antissepsia com solução de iodopovidona tópica e em anestesia local com xilocaína a 2% com vasoconstritor. Com o *laser* de CO_2_ na potência de 10 a 25W, utilizado de modo contínuo acoplado ao colposcópio, realizou-se incisão longitudinal com o feixe de *laser* com raio focalizado, de modo a abrir a cápsula do cisto. Os bordos laterais da incisão foram mantidos em tensão com pinças, para exposição da cavidade, com subsequente drenagem do conteúdo e lavagem do interior com solução salina estéril. Realizou-se a destruição do tecido capsular com vaporização, utilizando o feixe de *laser* desfocalizado (Figura 1). As pacientes foram orientadas a realizar banhos de assento com solução de iodopovidina diluída em água três vezes por dia, e abstinência sexual de 2 a 3 semanas. Antibióticos e analgésicos foram prescritos para pacientes que apresentavam sinais de infecção.

**Figura 1:** Procedimento a *laser* de CO2 no tratamento do cisto de Bartholin. (A) Exposição do cisto à direita, com infiltração com xilocaína a 2%. (B) Incisão com feixe *laser* focalizado sobre o cisto. (C) Abertura da cápsula com drenagem completa do conteúdo. (D) Reparo dos bordos com exposição da face interna da cápsula. (E) Vaporização da cápsula interna com *laser* raio desfocalizado

## RESULTADOS

O procedimento foi realizado com anestesia local em ambulatório. Todas as pacientes referiram desconforto mínimo, inerentes a procedimentos ambulatoriais, com dor à infiltração do anestésico e sensação de calor local com a aplicação do feixe *laser*.

As pacientes foram avaliadas 15 e 30 dias após o tratamento com *laser*. Nas 2 primeiras semanas, houve drenagem contínua de muco e, ao final do primeiro mês, cicatrização completa da ferida operatória.

Cinco pacientes (16%) apresentaram recidiva do cisto de Bartholin até 6 meses após primeira sessão de *laser* e foram submetidas a uma segunda sessão. Após a segunda fase de tratamento, não houve recidivas.

Nos 31 casos analisados, após período de cicatrização, não foi relatada dor no local da incisão e nem foram observadas cicatrizes e retrações.

## DISCUSSÃO

Na Itália, esta técnica tem sido utilizada por vários autores, devido à facilidade de acesso à tecnologia a *laser*.^8,16,17^ No nosso meio, observa-se pouca experiência de uso do *laser* para marsupialização do cisto de Bartholin, pelo custo do equipamento.^5^ Nosso serviço, voltado principalmente ao ensino da área médica, é considerado referência no atendimento de doenças do trato genital inferior. A experiência em tal técnica foi devido ao fato de termos o equipamento a *laser*, e à grande demanda de pacientes com a afecção em questão. Em análise anterior dos nossos casos, em 2007, já observávamos respostas positivas com esse método cirúrgico.^18^

A taxa de recidiva do nosso levantamento foi semelhante aos dados da literatura de acordo com as várias técnicas inicialmente descritas. O tratamento com *laser* de CO_2_ para cisto de Bartholin se mostra eficaz para resolução da doença e das comorbidades pós-tratamento, como dor e cicatrizes na região abordada.^18^ É um tratamento que associa a marsupialização com a destruição da cápsula doente pelo feixe de *laser*, oferecendo bons resultados, porém não diferindo daqueles que utilizam só a cirurgia ou a cauterização.^1,3,6,16^

O procedimento foi realizado ambulatorialmente, com o mínimo de sangramento durante o ato, além de boa e rápida cicatrização. Métodos cirúrgicos a bisturi frio não são isentos de apresentar sangramentos maiores, e os destrutivos com cauterização por meio de álcool ou nitrato de prata podem ser mais dolorosos no pós-operatório. Estes são fatores que têm influenciado nossa preferência pela técnica apresentada.^1,3,16^

O *laser* de CO_2_ mostrou ser método efetivo e com pouca morbidade. Observamos, com esta técnica, que a cicatrização foi rápida, com mínima fibrose e pouca dor remanescente, complicação foi observada pelas técnicas convencionais descrita por outros autores.^3,4,5^

Estas mulheres, em geral jovens, são sexualmente ativas e devemos nos preocupar com o frequente risco de sequela, como a dispareunia, consequente à fibrose da glândula, principalmente nas abordagens destrutivas de cauterização.

Além do mais, esta técnica permite novas sessões em caso de recidiva.

Por se tratar de método ambulatorial, a paciente não necessita se ausentar por muito tempo de suas atividades, não requerendo internação, e existe redução dos custos hospitalares. Por outro lado, o equipamento é de alto custo e requer profissional habilitado para manipulá-lo.

Por não existir período de internação hospitalar, a paciente é liberada para casa logo após o procedimento e em pouco tempo retorna às suas atividades diárias.

## CONCLUSÃO

A abordagem do cisto de Bartholin com o *laser* de CO_2_, de acordo com a técnica descrita de abertura do cisto, drenagem do conteúdo e vaporização da cápsula, pode ser usada como tratamento conservador eficaz com baixas taxas de recidiva, complicações e grande satisfação das pacientes. Não requer internação hospitalar que representa uma forma de economia financeira e temporal. A técnica a ser empregada depende da habilidade do médico e da disponibildade do equipamento.
